# Editorial for the Topical Collection “SARS-CoV-2 Infection and COVID-19 Disease”

**DOI:** 10.3390/pathogens13030191

**Published:** 2024-02-21

**Authors:** Luis Martinez-Sobrido, Marta L. DeDiego

**Affiliations:** 1 Texas Biomedical Research Institute, San Antonio, TX 78227, USA; 2 Department of Molecular and Cell Biology, Centro Nacional de Biotecnología (CNB-CSIC), 28049 Madrid, Spain

A previously unknown coronavirus, named Severe Acute Respiratory Syndrome Coronavirus 2 (SARS-CoV-2), emerged in the city of Wuhan, China, in December 2019. Its emergence resulted in the global coronavirus disease 2019 (COVID-19) pandemic that has dramatically impacted human health, social, and economic activities around the world, with a magnitude not seen since the 1918 Spanish influenza pandemic. 

This Special Issue, entitled “SARS-CoV-2 infection and COVID-19 disease”, covers aspects related to immune responses, antibodies, prophylactic vaccines, therapeutic antivirals, viral diagnostic, viral pathogenesis, and viral evolution in five review articles, one communication, and fifteen original research articles. 

Three review articles and three original research manuscripts document immune responses during SARS-CoV-2 infection. A systematic review and meta-analysis studied a previously proposed connection between COVID-19 severity and the levels of vitamin D and the pro-inflammatory cytokines tumor necrosis factor alpha (TNF-α) and interleukin 6 (IL-6). The authors found that IL-6 is an independent prognostic factor towards COVID-19 severity and mortality, as IL-6 significantly increases the risk of morbidity with severe COVID-19 and even mortality after SARS-CoV-2 infection. In contrast, increased TNF-α levels did not correlate with the risk of COVID-19 severity, but significantly correlated with the risk of COVID-19 mortality. Furthermore, the authors did not find a correlation between levels of vitamin D in patients with severe and non-severe COVID-19, and vitamin D deficiency did not significantly increase the risk of mortality of COVID-19 patients [1].

In another review article, the authors investigated the importance of measuring T-cell responses against SARS-CoV-2. While neutralizing antibodies have been considered as a correlate of protection against SARS-CoV-2 infection and severe COVID-19, T-cell responses are also important in protecting against severe COVID-19 and contribute to protection by prophylactic vaccines. As such, T-cell responses offer a long-lived line of protection and, contrary to humoral responses that differ among SARS-CoV-2 strains, T-cell responses largely retain reactivity against different SARS-CoV-2 variants. Due to the importance of T-cell responses in protection against SARS-CoV-2 infection and COVID-19, including protection against viral variants in novel vaccine strategies, the authors highlight the importance of monitoring the T-cell response, a process that will become more routine and streamlined with the development of T-cell assays [2].

A final review article focused on the differences in immune response against SARS-CoV-2 in different age groups, from infancy to adolescence, in comparison with adults. They report the outcomes and complications of COVID-19 among these different age groups in relation to immunological status, in both infected and vaccinated individuals. The authors indicate that the immune response and the components involved are different in neonates, infants, children, adolescent, and adults, but the immunological differences are mirrored during SARS-CoV-2 infection and post vaccination. The authors outline the importance of understanding the immunological mechanisms involved in SARS-CoV-2 infection and vaccination for the rational design of better prophylactic vaccines [3].

One scientific research article investigated the role of pre-existing cross-reactive coronavirus memory in the antibody response to viral spike (S) and nucleocapsid (N) proteins following SARS-CoV-2 infection. The breadth of the reactivity of circulating antibodies, plasmablasts, and memory B cells (MBCs) was analyzed. The results show that acutely infected subjects generated strong IgG responses to the viral S protein, including the receptor binding domain (RBD), the conserved S2 region, and the N protein. Both mild and severe infection expanded IgG MBC populations reactive to the S protein of SARS-CoV-2 and other β-coronaviruses, indicating that the response to the S and N proteins of SARS-CoV-2 involves pre-existing MBC activation and adaptation to novel features of these viral proteins to shape the response to SARS-CoV-2 infection [4].

In a separate research article, inflammatory markers, coagulopathy profiles, and severity outcomes in young adults (18–40 years old) were analyzed in 359 patients with confirmed cases of COVID-19 in Hermosillo, Mexico. Blood samples obtained during the first two weeks after the onset of COVID-19 symptoms were analyzed to determine the neutrophil/lymphocyte ratio (NLR), the neutrophil/monocyte ratio (NMR), the lymphocyte/monocyte ratio (LMR), and the platelet/lymphocyte ratio (PLR), as well as the C-reactive protein (C-RP) levels. The results showed increases in the NMR, LMR, and C-RP associated with COVID-19 severity in patients. Additionally, obesity, arterial hypertension, and type-2 diabetes mellitus (T2DM) were associated with COVID-19 severity outcome. Moreover, the authors found that 9.1% and 30.3% of young adults presented a novel COVID-19-associated coagulopathy (CAC) marker and the risk of CAC, respectively. These parameters can be considered independent biomarkers, reflecting an enhanced inflammatory process related to COVID-19 prognosis [5].

Finally, in another scientific article, the authors investigate an individual case of SARS-CoV-2 reinfection in a young Marine enrolled in a longitudinal COVID-19 Health Action Response for Marines study. This Marine was positive by RT-PCR for SARS-CoV-2 upon routine sampling on study day 7, although he was asymptomatic at that time, and he cleared the infection within seven days. On day 46, he developed symptoms consistent with COVID-19 and tested positive by RT-PCR for SARS-CoV-2 again. Viral genome sequencing determined that the Marine’s day-7 sample was infected with SARS-CoV-2 lineage B.1.340, whereas day-46 and day-49 samples corresponded to SARS-CoV-2 B.1.1 infection. The authors concluded that the results were consistent with a reinfection event, and that individuals who have experienced asymptomatic infection with a SARS-CoV-2 strain may fail to generate effective or long-lasting immunity against other SARS-CoV-2 strains [6].

Three original research manuscripts and one review article focused on antibody responses to SARS-CoV-2 infection. In the research article entitled “SARS-CoV-2 Seroprevalence and Neutralizing Antibody Response after the First and Second COVID-19 Pandemic Wave in Croatia”, the authors analyzed the seroprevalence and neutralizing (NT) antibody responses in the general Croatian population after the first (May–July 2020) and second (December 2020–February 2021) waves of the COVID-19 pandemic. They evaluated different age groups using commercial ELISA and virus neutralization test (VNT). As expected, a significant difference in the overall seroprevalence rate was found after the first and second waves of the pandemic due to increased incidence frequencies after the second wave. Seropositive individuals were detected in all studied age groups, with the lowest prevalence of NT antibodies found in the youngest and the oldest groups. However, these age groups showed the highest median NT titers. In the other age groups, seropositivity varied and a significant weak positive correlation between binding antibody level (ELISA) and neutralization titers (VNT) was observed, indicating that SARS-CoV-2 NT antibody titers are age-related, with the highest NT activity seen in children under 10 years old and adults over 50 years old [7].

A prospective study measured the titers of anti-SARS-CoV-2 IgA and IgG in the breast milk of 28 vaccinated lactating mothers, sampled at day 30 and day 60 after the second dose of anti-SARS-CoV-2 Pfizer or Moderna mRNA-based vaccines. They assessed whether protective antibodies against SARS-CoV-2 could be provided via breast milk. Anti-RBD IgA and IgG were detected in all breast milk samples, both in Pfizer- and Moderna-vaccinated mothers, without significant differences between them. The anti-RBD IgA titers were approximately five times higher than the anti-RBD IgG titers. The anti-RBD IgA and IgG titers correlated with the infants’ age, but they were not correlated with vaccine type or mother’s age. This study concluded that anti-SARS-CoV-2 IgA and IgG can be found in the milk of mothers vaccinated with Pfizer or Moderna mRNA vaccines and should offer protection to the newborn, considering that the antibody titers did not decrease after 60 days [8].

In the manuscript entitled “GMP Manufacturing and IND-Enabling Studies of a Recombinant Hyperimmune Globulin Targeting SARS-CoV-2”, the authors generated a first-in-class recombinant hyperimmune globulin therapeutic against SARS-CoV-2 comprising 12,500 antibodies (GIGA-2050). Hyperimmune globulin drugs are traditionally manufactured from pooled immunoglobulins from vaccinated or convalescent donors, and have been used to treat SARS-CoV-2 infections. Here, GIGA-2050 was scaled-up for clinical manufacturing, and multiple lots were assessed for consistency. GIGA-2050 was purified and tested for good laboratory procedures (GLPs) toxicology, pharmacokinetics, and in vivo efficacy against natural SARS-CoV-2 infection in mice. Non-human primate toxicology and pharmacokinetics studies were also conducted to demonstrate that GIGA-2050 is safe and has a half-life similar to other recombinant human IgG1 antibodies. Altogether, this study demonstrates that GIGA-2050 can be used as a new class of drug for the treatment of SARS-CoV-2 infection [9].

Finally, in a review article published in August 2022, the authors focused on the therapeutic indications of currently available monoclonal antibodies and convalescent plasma for COVID-19 pre- and post-exposure prophylaxis. Early in the COVID-19 pandemic, the United States Food and Drug Administration (FDA) and the European Medical Agency (EMA) authorized SARS-CoV-2 monoclonal antibodies to treat mild to moderate COVID-19 in patients at risk of developing severe disease. Later, SARS-CoV-2 monoclonal antibodies were also authorized for primary and secondary prophylaxis in patients at high risk of severe disease. Primary or pre-exposure prophylaxis prevents COVID-19 in unexposed people, whereas secondary or post-exposure prophylaxis prevents COVID-19 in recently exposed people [10].

Two original research manuscripts relate to SARS-CoV-2 vaccination. In the first article, the SARS-CoV-2 S glycoprotein RBD, which allows virus binding to cells, was displayed on immunogenic liposomes (RBD-liposomes) and intranasally administered. The vaccination resulted in the production of antigen-specific IgA and antigen-specific cellular responses in the lungs of vaccinated mice. Transgenic K18 mice expressing the human angiotensin converting enzyme 2 (hACE2), the cellular receptor for SARS-CoV-2, were immunized intramuscularly or intranasally with RBD-liposomes and challenged with SARS-CoV-2. Vaccinated mice showed reduced viral titers compared with mock-vaccinated mice, with the intramuscular route achieving complete viral clearance. Importantly, both vaccine administration routes led to full protection against lethal viral infection, demonstrating the potential of immunization with liposome-displayed antigen vaccines for the prevention of SARS-CoV-2 infection [11].

In the second vaccine study, the authors generated a whole-virus SARS-CoV-2 vaccine using a novel psoralen inactivation method (SARS-CoV-2 PsIV) and evaluated its immunogenicity in mice using two different adjuvants, alum, and Advax-2. A comparison between the immunogenicity of SARS-CoV-2 PsIV against SARS-CoV-2 DNA vaccines expressing either full-length or truncated S protein was evaluated. The SARS-CoV-2 PsIV against a DNA prime and a SARS-CoV-2 PsIV boost regimen was also compared. After two doses, the SARS-CoV-2 PsIV, when administered with alum or Advax-2 adjuvants, generated dose-dependent neutralizing antibody responses in mice. The pattern of cytokine ELISPOT responses to antigen stimulation observed in this study indicated that SARS-CoV-2 PsIV with the alum adjuvant promoted a Th2-type response, while SARS-CoV-2 PsIV with the Advax-2 adjuvant promoted a Th1-type response. These results demonstrated the feasibility of implementing SARS-CV-2 PsIV as a safe and effective vaccine to protect against SARS-CoV-2 infection [12].

Two research manuscripts and one communication focused on antivirals against SARS-CoV-2. In the first research document, the authors evaluate the immunomodulatory effects of viable and non-viable *Lactiplantibacillus plantarum* (*L. plantarum*) strains, and the capacity of these immunobiotic lactobacilli to reduce susceptibility to SARS-CoV-2 infection in human respiratory epithelial Calu-3 cells. Immunobiotic *L. plantarum* strains MPL16 and CRL1506 differentially modulated the expression of interferons (IFNs), inflammatory cytokines, and IFN-stimulated genes (ISGs) in Calu-3 cells stimulated with the TLR3 agonist poly(I:C). Furthermore, MPL16 and CRL1506 strains increased the resistance of Calu-3 cells to SARS-CoV-2 infection, with MPL16 being more protective than CRL1506. However, neither non-viable MPL16 or CRL1506 immunomodulatory strains, or CRL1905 or MPL18 non-immunomodulatory strains affected the resistance of Calu-3 cells to SARS-CoV-2 infection or the immune response to poly(I:C). These results suggest the potential beneficial effects of immunomodulatory probiotics on SARS-CoV-2 infection. Unfortunately, mechanistic studies and validation experiments in animal models of SARS-CoV-2 infection were not conducted to extrapolate initial in vitro to in vivo immunobiotic studies [13].

Similarly to previous research, a communication study performed by the same group evaluated the immunomodulatory effects of the *Dolosigranulum pigrum* strain 040417 and its ability to inhibit SARS-CoV-2 replication in human respiratory epithelial cells. The results demonstrate that the *Dolosigranulum pigrum* strain 040417 differentially modulated the production of IFNs and inflammatory cytokines in the culture supernatants of Calu-3 cells stimulated with poly(I:C) or infected with SARS-CoV-2, and reduced SARS-CoV-2 replication and cellular damage after viral infection. However, the *Dolosigranulum pigrum* strain 030918 was not able to inhibit SARS-CoV-2 infection, indicating a strain-specific immunomodulatory effect for respiratory commensal bacteria. These results indicate the feasibility of certain *Dolosigranulum pigrum* strains as a promising alternative for combating SARS-CoV-2 infection and reducing the severity of COVID-19 [14].

In the last antiviral manuscript of the Special Issue, the authors assessed the antiviral activity of 21 plant-based ingredients, including glycyrrhizin, withanone, aloe-emodin, rhein, emodin, chrysophanol, physcion, kaempferol, pogallin A, gallic acid, naringin, quercetin, luteolin, and apigenin. They tested the ingredients against SARS-CoV-2 using pseudotyped SARS-CoV-2 in a lentiviral delivery system in human HEK293T cells. The researchers identified that the natural extracts in a herb-derived phytoconstituent-based formulation, BITS-003, had strong virucidal properties. BITS-003 effectively inactivated viruses as well as bacteria and yeast, suggesting that topical use of the BITS-003 formulation as a mouthwash/gargle could be effective in reducing symptoms caused by respiratory viral infections, and possibly in decreasing viral loads in the buccal/oral cavity [15].

Two original research articles focused on SARS-CoV-2 diagnosis. In the first manuscript, the authors compared the sensitivity of tests for various sample specimens using nasopharyngeal swabs, oropharyngeal swabs, oracol-collected saliva, throat washes, and rectal samples from 75 confirmed COVID-19-positive patients. Sampling was repeated after 7 to 10 days, and then after 14 to 20 days to perform a longitudinal analysis of sample specimen sensitivity. At the first time point, the percentages of SARS-CoV-2-positive samples were 84.3%, 74%, 68.2%, 58.8%, and 3.5% for nasopharyngeal, throat washing, oracol-collected saliva, oropharyngeal, and rectal samples, respectively. However, the sensitivity of all sampling methods, except for throat wash samples, decreased rapidly at later time points compared with the first collection time point. The throat washing method exhibited higher sensitivity than the gold standard nasopharyngeal swab at the second and third time points after the first positive test date, whereas the nasopharyngeal swabs were the most sensitive specimens for early detection after symptom onset. These results suggest that throat washing is a more sensitive alternative method for SARS-CoV-2 diagnosis, and SARS-CoV-2 persists longer in the throat and saliva than in the nasopharynx [16].

In the second research article, the authors evaluated the performance of a multiplex reverse transcriptase real-time PCR (RT-rPCR) assay specific to seven human pathogenic coronaviruses in detecting SARS-CoV-2 directly from nasopharyngeal swabs obtained from suspected COVID-19-infected patients. The assay also simultaneously detected different human pathogenic coronaviruses if present. From 1195 clinical samples suspected of COVID-19 infection, the assay identified that 69% of the samples tested positive for SARS-CoV-2, a result that was confirmed using a different SARS-CoV-2 RT-PCR kit. Importantly, none of these clinical samples were positive for the other coronaviruses tested, including SARS-CoV, MERS-CoV, or human coronavirus (HCoV), suggesting that during the endemic phase of COVID-19, infection with other HCoV was very uncommon. This study also demonstrated that the multiplex RT-rPCR is time- and cost-saving, easy to implement, and a sensitive assay for detecting SARS-CoV-2 and other HCoVs that could affect public health [17].

Two documents were associated with SARS-CoV-2 pathogenicity. In the review article entitled “Post COVID-19 syndrome in patients with asymptomatic/mild form”, the authors evaluated post COVID-19 syndrome (PCS) in publications available from online databases from December 2019 to September 2021. The researchers found that, on average, PCS developed in 30–60% of patients, mainly among women. The most common PCS included fatigue, shortness of breath, cough, and anosmia. Notably, the authors found that gender (female) and the presence of anosmia during an asymptomatic or mild course of COVID-19 can be predictive for the development of PCS, which can be caused by autoimmune damage to neurons, glia, and cerebral vessels [18].

In a separate research article, the authors investigate the effect of bioaerosols on the seasonality of influenza-like illnesses, including COVID-19 in Chicago. The authors found that the end of the flu season coincides with the beginning of pollen season, as well as the onset of the seasonal aerosolization of mold spores. The researchers suggest that bioaerosols, especially mold spores, compete with viruses for a shared receptor. They further suggest that the periodicity of influenza-like illnesses, including COVID-19, is a consequence of seasonal factors that influence the aerosolization of competing species [19].

Two original research articles focused on the evolution of SARS-CoV-2. In the first manuscript, the authors characterize the mutant spectrum of hepatitis C virus (HCV) and SARS-CoV-2 at the nucleotide and amino acid levels. The authors concluded that the number of different mutations found at low frequency in SARS-CoV-2 variants increases dramatically as the cut-off frequency for mutation detection is lowered from 0.5% to 0.1%. The authors also demonstrate that contrary to HCV, SARS-CoV-2 mutant spectra exhibit a deficit of intermediate frequency amino acid substitutions, suggesting that the possible origins and implications of mutant spectra differ among RNA viruses [20].

In the second study, the authors assess the impact of mutations in the SARS-CoV-2 genome on the clinical phenotype and associated comorbidities, aspects which are important for treatment and prevention in the progress of the COVID-19 pandemic. The authors analyzed the association between the clinical subphenotypes and genomic mutations with respect to the severity and outcome of the patients. They found a significant association between the requirement of respiratory support and comorbidities. The researchers also identified six mutations in the viral genome that were significantly correlated with severity and mortality. Structural alterations at the RNA and protein levels of three of the identified mutations present in the ORF3a and nucleocapsid (N) proteins suggest that the RNA secondary structure can be one of the modulators of disease outcomes. These findings demonstrate the importance of genomic surveillance to identify individuals needing priority medical support [21].

We believe that the manuscripts published in this Special Issue represent some of the most current advances related to research on SARS-CoV-2 infection and COVID-19. We have endeavored to highlight notable review and original research articles in this Special Issue to encourage other investigators to conduct future studies aiming to understand SARS-CoV-2 infection and pathogenesis, epidemiology and evolution, vaccines, antivirals, neutralizing antibodies, and immune responses. Moreover, we hope that the manuscripts in this Special Issue open the door to collaborations, with the goal of improving the development of efficient prophylactic and therapeutic approaches for the efficient control of SARS-CoV-2 infection and the ongoing COVID-19 pandemic.

Finally, we would like to thank all the authors and their respective institutions that contributed to this Special Issue, for their participation and for taking the time to submit manuscripts. Likewise, we want to also thank the Editorial Office of *Pathogens* for their help, guidance, and assistance in putting together this Special Issue.
